# Efficacy and safety of anlotinib for triple-negative breast cancer with brain metastases

**DOI:** 10.3389/fonc.2024.1439984

**Published:** 2024-10-03

**Authors:** Zeyu Liu, Ming Li, Ziyi Zhao, Aina Liu, Ping Sun

**Affiliations:** ^1^ Department of Oncology, The Affiliated Yantai Yuhuangding Hospital of Qingdao University, Yantai, China; ^2^ Department of Hand and Foot, Microsurgery, The Affiliated Hospital of Qingdao University, Qingdao, China

**Keywords:** brain metastases, triple-negative breast cancer, anlotinib, tyrosine kinase inhibitor, antiangiogenesis, side effect

## Abstract

**Background:**

The anti-angiogenic agent anlotinib offers a new treatment option for triple-negative breast cancer (TNBC) patients with brain metastases. This study aimed to evaluate the efficacy and safety of anlotinib in the treatment of TNBC patients with brain metastases.

**Methods:**

Between October 2019 and April 2024, 29 TNBC patients with brain metastases who had failed prior therapy and were treated with anlotinib were retrospectively analyzed. The primary endpoint was central nervous system (CNS) progression-free survival (PFS), and secondary endpoints included overall survival (OS), intracranial disease control rate (iDCR), intracranial objective response rate (iORR), and safety.

**Results:**

The median CNS PFS of 29 patients was 7.2 months (95% confidence interval [CI], 3.5-10.9 months), and the median OS was 10.2 months (95% CI, 5.6-14.8 months). The iORR and iDCR were 31.0% and 86.2%, respectively. Five patients (17.2%) experienced grade 3-4 adverse events (AEs), with bone marrow suppression (2/29, 6.9%) being the most common. Most AEs were clinically manageable, and no treatment-related death was observed.

**Conclusion:**

Anlotinib demonstrated encouraging efficacy and manageable toxicity in the treatment of TNBC patients with brain metastases who had failed standard treatment.

## 1 Introduction

Breast cancer remains the most prevalent form of cancer in women and is the second most common cause of the development of brain metastases after lung cancer (1, 2). Triple-negative breast cancer (TNBC) accounts for 15-20% of all breast cancer cases. Compared with other subtypes, metastases from TNBC are more common with a worse prognosis. Since patients with TNBC are unable to benefit from endocrine therapy or human epidermal growth factor receptor 2 (HER2)-targeted therapy, the standard of care for nonsurgical TNBC remains nonspecific chemotherapy. In addition to chemotherapeutic agents such as paclitaxel and cisplatin, the main treatments for TNBC include immunotherapeutic agents targeting PD-L1 such as atezolizumab and pembrolizumab (3) Thus, more effective therapies for TNBC remain to be developed. With the prolonged overall survival (OS) of patients with advanced breast cancer, the incidence of brain metastases has increased remarkably. Brain metastases occur in about 25% of patients with advanced breast cancer and even up to 40% of patients with TNBC, greatly influencing the quality of life of patients (4, 5).

The blood-brain barrier hinders the entry of numerous drugs into the brain, limiting the efficacy of drug therapy in patients with brain metastases (6). As a consequence of these constraints, radiotherapy or surgical interventions are typically utilized for treating patients with brain metastases. Therapies utilizing drugs for controlling brain metastases are still being investigated. Although traditional chemotherapy has not yielded optimal results in addressing brain metastases, tyrosine kinase inhibitors (TKIs) targeting vascular endothelial growth factor (VEGF) have proved to be efficacious in controlling brain metastases.

Neovascularization is a key step in the proliferation of malignant tumors, making anti-angiogenesis an important strategy in anti-tumor therapy. Tumor growth requires an adequate blood supply to provide nutrients. It has been shown that angiogenesis not only affects tumor growth but also is an important factor in promoting tumor metastases. Thus, it is possible to inhibit tumor growth by suppressing angiogenesis (7, 8). The growth and metastasis of breast cancer are dependent on angiogenesis, and the expression of VEGF in breast cancer tissues far exceeds that in normal tissues (9). Moreover, TNBC had significantly higher VEGF expression levels than patients with non-TNBC. VEGF secreted by breast cancer tissues acts on vascular endothelial growth factor receptor (VEGFR), which promotes the division and proliferation of vascular endothelial cells, and induces tumor angiogenesis, thus providing sufficient blood supply for breast cancer progression. Bevacizumab is an anti-angiogenic agent that is commonly used in metastatic breast cancer. Previous studies have shown that bevacizumab improves central nervous system (CNS) objective response rate (ORR) and median progression-free survival (PFS) in patients with brain metastases from breast cancer, but along with a high risk of grade 3-4 toxicity (10, 11).

Anlotinib is a novel small-molecule TKI with anti-angiogenic properties and the ability to cross the blood-brain barrier. It targets more sites than bevacizumab and exhibits better systemic efficacy than bevacizumab. The primary targets of anlotinib include VEGFR, fibroblast growth factor receptor (FGFR), platelet-derived growth factor receptor (PDGFR), and stem cell growth factor receptor (c-Kit) (12–14). Anlotinib can reduce the levels of proangiogenic factors and enhance the expression of immune cell adhesion molecules, chemokines, and their receptors. Additionally, it can effectively impede both tumor angiogenesis and growth (12). As a result, it exhibits encouraging efficacy in anti-tumor therapy with minimal toxicity. Besides, anlotinib is administered orally and is therefore more acceptable to patients than bevacizumab, without the risk of injections. A phase II study has established the positive impact of anlotinib in addressing HER2-negative advanced or metastatic breast cancer (15). Furthermore, anlotinib also shows promising efficacy in brain metastases from lung cancer (16). Recently, it has been observed to be effective in treating TNBC patients with brain metastases. In this context, a retrospective study was conducted to illustrate brain metastases from TNBC. The results showed that anlotinib yielded outstanding anti-tumor activity without inducing severe treatment-related adverse events (AEs).

## 2 Methods

### 2.1 Study design and patients

This was a single-center retrospective study conducted in The Affiliated Yantai Yuhuangding Hospital of Qingdao University. From October 2019 to April 2024, TNBC patients with brain metastases who had received second-line or subsequent treatment with anlotinib at our hospital were included and analyzed for efficacy and safety. Eligible patients were 30-70 years old and had an Eastern Cooperative Oncology Group (ECOG) performance status of 0-2. Patients with leptomeningeal disease were not included in this study. Steroids were used in some patients due to severe cases of cerebral edema. Patients who have been previously submitted to CNS radiotheraphy for more than six months and have experienced progression of intracranial lesions will be included in the study. The patterns of technique used in terms of radiotheraphy depends on the patient’s intracranial lesions; patients with 1-2 intracranial lesions were treated with Stereotactic Radio-Surgery (SRS), patients with more than 5 intracranial lesions were treated with Whole Brain Radiotheraphy (WBRT), and patients with 3-5 intracranial lesions were given the option of using either SRS or WBRT, depending on the specifics of the lesions.

### 2.2 Treatments

All patients received anlotinib monotherapy or in combination with chemotherapy. The primary chemotherapeutic agents included capecitabine (1000 mg/m^2^ twice daily for 14 days followed by 7 days of rest), gemcitabine (1000 mg/m^2^ on days 1 and 8), nab-paclitaxel (125 mg/m^2^ on days 1 and 8), vinorelbine (25 mg/m^2^ on days 1 and 8), and eribulin (1.4 mg/m^2^ on days 1 and 8). Anlotinib was administered at a dosage of 12 mg, 10 mg, or 8 mg orally on days 1-14 of a 21-day cycle. The recommended dosage for anlotinib was 12 mg. When the patient experienced AEs of grade 2 or higher, anlotinib was suspended, resumed when the AEs dropped below grade 2, and the dose of anlotinib was adjusted downward to 10 mg, and when this event occurred a second time, the dose of anlotinib was adjusted downward to 8 mg.

### 2.3 Efficacy and safety assessments

Efficacy was assessed every two cycles according to the Response Evaluation Criteria in Solid Tumors version 1.1 (RECIST 1.1). Response to treatment was categorized as complete response (CR), partial response (PR), stable disease (SD), and progressive disease (PD). The primary endpoint was CNS PFS, which was defined as the time from the start of treatment to intracranial disease progression or death. Secondary endpoints included OS (the time from the start of treatment to death from any cause), intracranial ORR (iORR; the proportion of patients who had an intracranial PR [iPR] or iCR at the best response), intracranial disease control rate (iDCR; the proportion of patients who had an iPR, iCR, or iSD at the best response), and safety.

### 2.4 Statistical analysis

All data were statistically analyzed using SPSS version 27.0 (IBM, New York, USA). PFS and OS were estimated by the Kaplan-Meier method. The effect of clinical factors on PFS and OS was examined by a stratified Cox proportional hazards regression model. The significance level was set at *p*<0.05.

## 3 Result

### 3.1 Patient characteristics

The median age of the 29 patients was 57 years (95% CI, 52.3-59.5 years). The majority of patients were younger than 60 years of age (19/29, 65.5%), had an ECOG performance status of 0-1 (24/29, 82.8%), had multiple brain metastases (16/29, 55.2%), had ≥3 metastatic sites (21/29, 72.4%), and received anlotinib as third-line or subsequent treatment (20/29, 69.0%). The clinical characteristics of the 29 patients are summarized in **Table 1**.

**Table 1:** Clinical characteristics of the study population.

Clinical characteristic	N=29
Age of enrollment (years; n, %)	
<60	19 (65.5)
≥60	10 (34.5)
ECOG performance status (n, %)	
0-1	24 (82.8)
2	5 (17.2)
Number of brain metastases (n, %)	
Single	13 (44.8)
Multiple (2) Multiple (3-5) Multiple (>5)	8 (27.6) 7(24.1) 1(3.5)
Number of metastatic sites (n, %)	
1-2	8 (27.6)
≥3	21 (72.4)
Current treatment line	
1-2	9 (31.0)
≥3	20 (69.0)
Prior chemotherapy after metastases (n, %)	
No	11 (37.9)
Yes	18 (62.1)
Prior anti-angiogenesis therapy after metastases (n, %)	
No	23 (79.3)
Yes	6 (20.7)
Intracranial radiotherapy (n, %)	
No	12 (41.4)
Yes	17 (58.6)
Combined with chemotherapy (n, %)	
No	14 (48.3)
Yes	15 (51.7)
Symptomatic brain metastases (n, %)	
No	19 (65.5)
Yes	10 (34.5)
Anlotinib monotherapy (n, %)	
No	15 (51.7)
Yes	14 (48.3)

ECOG, Eastern Cooperative Oncology Group. The median number of BM is 2.5 (95% CI, 2.3-3.6).

### 3.2 Efficacy

**Figure 1:**
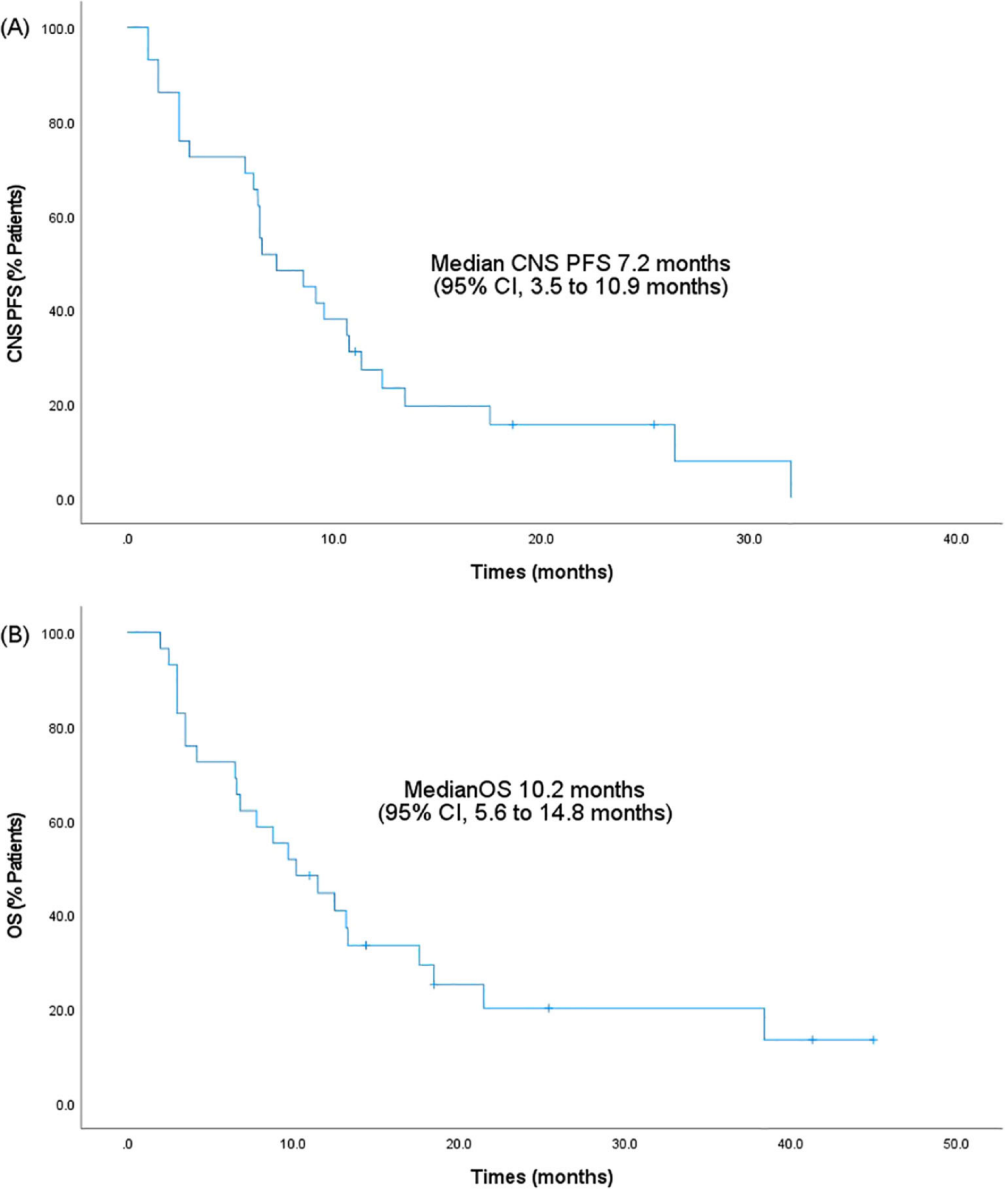
Kaplan-Meier survival analysis. **(A)** CNS PFS. **(B)** OS. PFS, progression-free survival; 95% CI, 95% confidence intervals; OS, overall survival; CNS, central nervous system.

**Figure 2:**
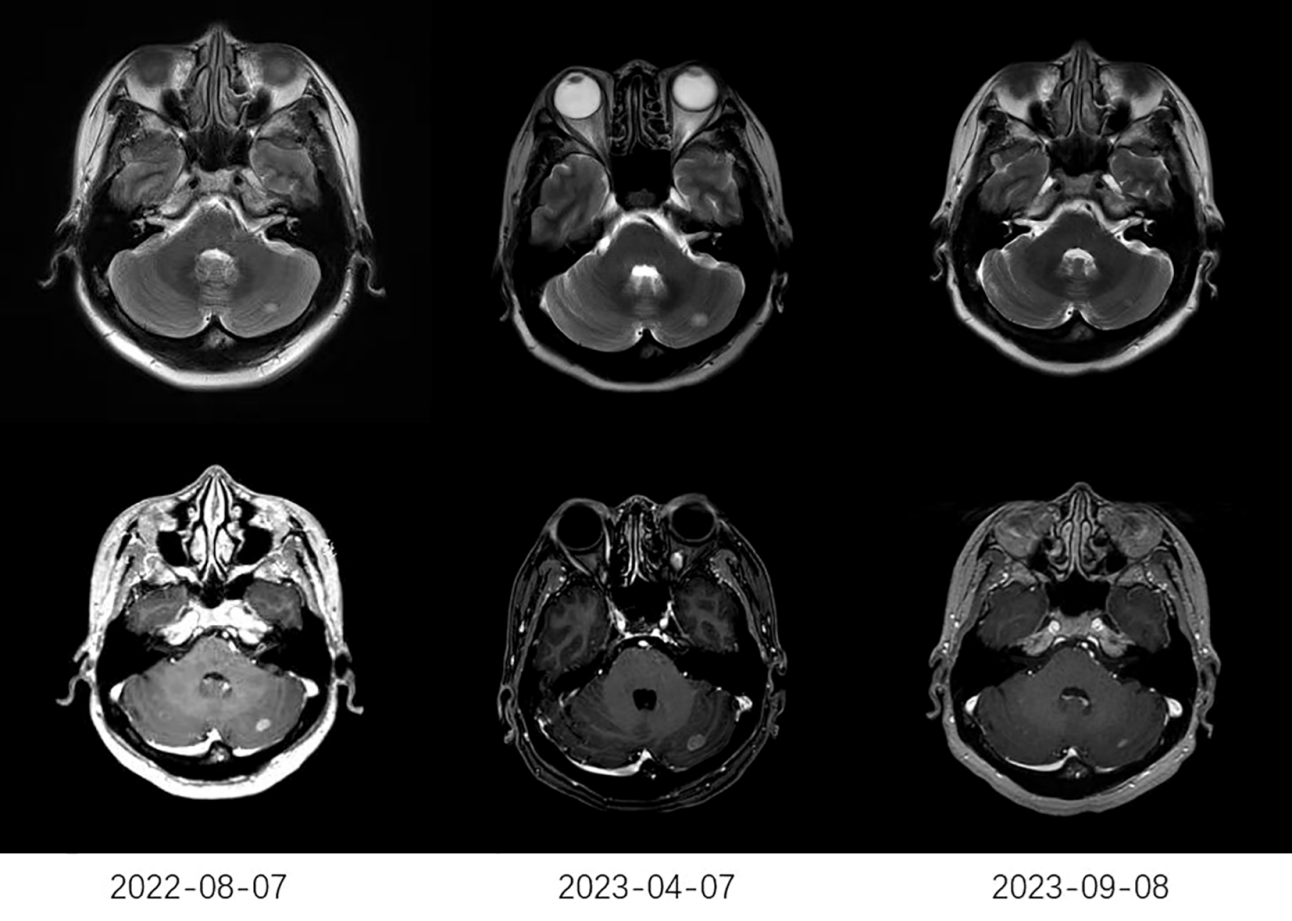
The typical MRI images of one patient. The MRI on April 7, 2023, showed a larger lesion than that on August 7, 2022. After receiving anlotinib (2023-09-08), MRI showed a significant reduction in the lesion compared to that before receiving anlotinib (2023-04-07). MRI, typical magnetic resonance imaging.

The treatment response to anlotinib is summarized in **Table 2**. The median CNS PFS of 29 patients was 7.2 months (95% confidence interval [CI], 3.5-10.9 months; **Figure 1A**), and the median OS was 10.2 months (95% CI, 5.6-14.8 months; **Figure 1B**). Systemic ORR and DCR were 34.5% and 82.8%, respectively. Among the 29 patients, 1 achieved an iCR, 8 achieved an iPR, 16 had iSD, and 4 had iPD, with an iORR of 31.0% and an iDCR of 86.2%. In addition, Among 16 patients who had iSD, 9 patients had multiple BMs, 7 patients had single BM, 2 patients had received prior antinagiogenic therapies. Among 8 patients who had iPR, 3 patients had multiple BMs, 5 patients had single BM, 1 patient had received prior antinagiogenic therapies. The patient with iCR had multiple BMs and had not received prior antinagiogenic therapies. **Figure 2** shows the typical and clear magnetic resonance imaging (MRI) images of one patient before and after the administration of anlotinib. This patient has never been previously submitted to CNS radiotheraphy treatment.

**Table 2:** Treatment response based on RECIST 1.1.

	All patients (n=29)
Best systemic response (n, %)	
CR	1 (3.4)
PR	9 (31.0)
SD	14 (48.3)
PD	5 (17.2)
ORR	10 (34.5)
DCR	24 (82.8)
Best intracranial response (n, %)	
iCR	1(3.4)
iPR	8(27.6)
iSD	16(55.2)
iPD	4(13.8)
iORR	9(31.0)
iDCR	25(86.2)

RECIST 1.1, Response Evaluation Criteria in Solid Tumors version 1.1; CR, complete response; PR, partial response; SD, stable disease; PD, progressive disease; ORR, objective response rate; DCR, disease control rate; iCR, intracranial CR; iPR, intracranial PR; iSD, intracranial SD; iPD, intracranial PD; iORR, intracranial ORR; iDCR, intracranial DCR.

### 3.3 Prognostic factors for survival

A stratified Cox proportional hazards regression model was performed to assess the effect of different clinical factors on CNS PFS and OS. All variables that were significant at *p*<0.05 in univariate analysis were included in the multivariate analysis (**Tables 3**, **4**). ECOG performance status, treatment regimens, and prior anti-angiogenesis therapy after metastases were statistically significant in the univariate analysis for the CNS PFS (all *p*<0.05). After including the above clinical factors in the multivariate analysis, ECOG performance status of 2 (hazard ratio [HR]=4.8; 95% CI, 1.3-17.5; *p*=0.02) and prior anti-angiogenesis therapy after metastases (HR=4.4; 95% CI, 1.4-13.5; *p*=0.01) were proved to be independent and meaningful unfavorable prognostic factors for the CNS PFS. In the univariate analysis for the OS, treatment regimens, prior anti-angiogenesis therapy after metastases, and symptomatic brain metastases were estimated to be statistically significant (all *p*<0.05). After including the above clinical factors in multivariate analysis, prior anti-angiogenesis therapy after metastases (HR=4.3; 95% CI, 1.3-14.1; *p*=0.02) and symptomatic brain metastases (HR=4.2; 95% CI, 1.5-12.1; *p*=0.01) were independent and meaningful prognostic factors for the OS.

**Table 3:** Univariate and multivariate analyses for the CNS PFS.

Variables	Univariate analysis		Multivariate analysis	
	HR (95% CI)	P value	HR (95% CI)	P value
Age of enrollment years (≥60/<60)	0.7 (0.3-1.5)	0.3		
Current treatment line (≥3/1-2)	1.8 (0.7-4.4)	0.2		
ECOG performance status (2/0-1)	3.6 (1.2-11.2)	0.02	4.8 (1.3-17.5)	0.02
Number of metastatic sites (≥3/1-2)	0.7 (0.3-1.6)	0.4		
Number of brain metastases (multiple/single)	2.1 (0.9-4.9)	0.1		
Symptomatic brain metastases (yes/no)	2.2 (1.0-5.4)	0.05		
Prior chemotherapy after metastases (yes/no)	0.7 (3.1-1.6)	0.4		
Prior anti-angiogenesis therapy after metastases (yes/no)	3.2 (1.2-8.6)	0.02	4.4 (1.4-13.5)	0.01
Intracranial radiotherapy (yes/no)	1.0 (0.4-2.2)	0.9		
Combined with chemotherapy (yes/no)	0.4 (0.2-0.8)	0.01	0.8 (0.04-16.2)	0.9
Anlotinib monotherapy (yes/no)	2.6 (1.2-6.0)	0.02	1.6 (0.1-32.6)	0.8

HR, hazard ratio; PFS, progression-free survival; 95% CI, 95% confidence intervals; ECOG, Eastern Cooperative Oncology Group; CNS, central nervous system.

**Table 4:** Univariate and multivariate analyses for the OS.

Variables	Univariate analysis		Multivariate analysis	
	HR (95% CI)	P value	HR (95% CI)	P value
Age of enrollment years (≥60/<60)	0.7 (0.3-1.6)	0.4		
Current treatment line (≥3/1-2)	2.1 (0.8-5.4)	0.1		
ECOG performance status (2/0-1)	2.0 (0.7-6.0)	0.2		
Number of metastatic sites (≥3/1-2)	1.4 (0.5-3.5)	0.5		
Number of brain metastases (multiple/single)	1.9 (0.8-4.5)	0.1		
Symptomatic brain metastases (yes/no)	3.0 (1.2-7.4)	0.02	4.2 (1.5-12.1)	0.01
Prior chemotherapy after metastases (yes/no)	1.1 (0.5-2.6)	0.8		
Prior anti-angiogenesis therapy after metastases (yes/no)	2.7 (1.0-7.4)	0.05	4.3 (1.3-14.1)	0.02
Intracranial radiotherapy (yes/no)	1.1 (0.5-2.6)	0.8		
Combined with chemotherapy (yes/no)	0.4 (0.2-0.9)	0.03	1.9 (0.1-26.7)	0.6
Anlotinib monotherapy (yes/no)	2.5 (1.0-6.2)	0.04	0.3 (0.3-42.8)	0.3

HR, hazard ratio; OS, overall survival; 95% CI, 95% confidence intervals; ECOG, Eastern Cooperative Oncology Group.

**Figure 3:**
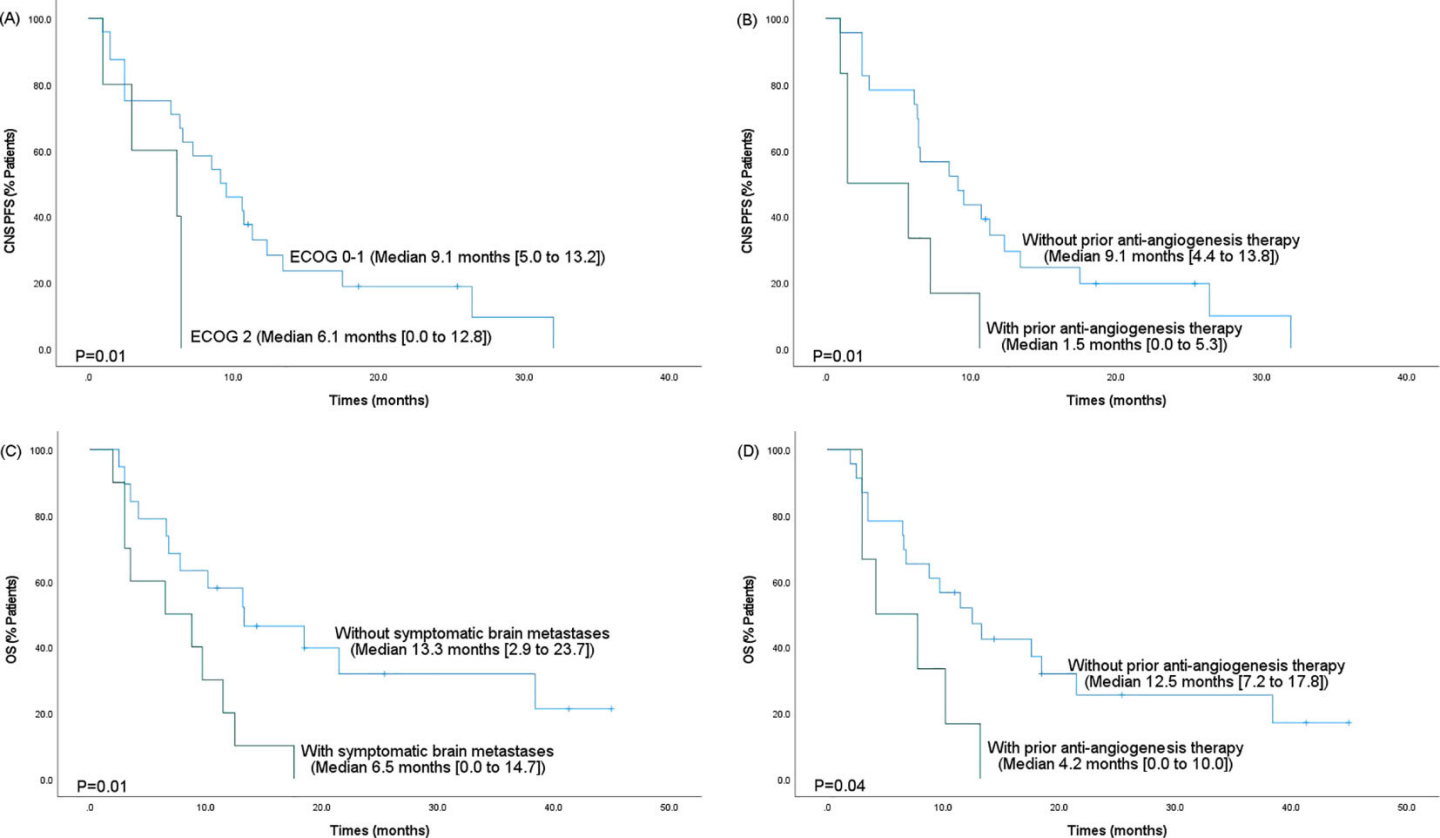
Kaplan-Meier survival analysis for the CNS PFS and OS based on significant predictors in the multivariate analysis. **(A)** The CNS PFS based on ECOG performance status. **(B)** The CNS PFS based on prior anti-angiogenesis therapy after metastases. **(C)** The OS based on the presence of symptomatic brain metastases. **(D)** The OS based on prior anti-angiogenesis therapy after metastases. PFS, progression-free survival; 95% CI, 95% confidence intervals; OS, overall survival; CNS, central nervous system.

Kaplan-Meier survival analysis was performed based on significant predictors in the multivariate analysis. As shown in **Figure 3**, shorter CNS PFS was more likely to occur in patients with an ECOG performance status of 2 (6.1 vs. 9.1 months, *p*=0.01) or in patients who had received prior anti-angiogenesis therapy after brain metastases development (1.5 vs. 9.1 months, *p*=0.01). Besides, the OS was significantly shorted in cases with symptomatic brain metastases (6.5 vs. 13.3 months, *p*=0.01) or in cases who had received prior anti-angiogenesis therapy after metastases (4.2 vs. 12.5 months, *p*=0.04).

### 3.4 Safety

AEs that occurred during treatment with anlotinib in the 29 patients were summarized in **Table 5**. The most common AEs of all grades were bone marrow suppression (8/29, 27.6%), fatigue (7/29, 24.1%), hypertension (6/29, 20.7%), gastrointestinal response (6/29, 20.7%), and hand-foot syndrome (6/29, 20.7%). A total of 5 patients (17.2%) experienced grade 3-4 AEs. The most common grade 3-4 AE was bone marrow suppression (2/29, 6.9%). No treatment-related death was observed.

**Table 5:** Adverse events that occurred during treatment with anlotinib in 29 patients.

Treatment-related adverse events	All grades (n, %)	Grade 1-2 (n, %)	Grade 3-4 (n, %)
Bone marrow suppression	8 (27.6)	6 (20.7)	2 (6.9)
Fatigue	7 (24.1)	7 (24.1)	0 (0)
Hypertension	6 (20.7)	5 (17.2)	1 (3.4)
Gastrointestinal response	6 (20.7)	5 (17.2)	1 (3.4)
Hand-foot syndrome	6 (20.7)	5 (17.2)	1 (3.4)
Anorexia	5 (17.2)	5 (17.2)	0 (0)
Hepatic dysfunction	4 (13.8)	4 (13.8)	0 (0)
Proteinuria	4 (13.8)	4 (13.8)	0 (0)
Mucositis oral	4 (13.8)	4 (13.8)	0 (0)
Thyroid dysfunction	4 (13.8)	4 (13.8)	0 (0)
Rash	3 (10.3)	3 (10.3)	0 (0)
Hemoptysis	3 (10.3)	3 (10.3)	0 (0)
Anemia	2 (6.9)	2 (6.9)	0 (0)
Pneumonitis	1 (3.4)	1 (3.4)	0 (0)

## 4 Discussion

The presence of brain metastases from breast cancer is associated with a poor prognosis, and the incidence of this complication has been increasing in recent years (4). Given the challenge of penetrating the blood-brain barrier with traditional chemotherapeutic drugs, prevailing treatment options for brain metastases mainly include surgical interventions and radiotherapy. Exploration of drug-based therapies for optimal and sustained control of brain metastases remains a subject of ongoing research.

Anti-angiogenic agents represented by bevacizumab have shown promising efficacy in the treatment of brain metastases from breast cancer, suggesting that anti-angiogenesis therapy can inhibit the progression of brain metastases (10). Meanwhile, previous studies have demonstrated the favorable efficacy of anti-angiogenic agents such as bevacizumab and apatinib in TNBC (17, 18). However, the incidence of AEs related to bevacizumab is high and bevacizumab has difficulty crossing the blood-brain barrier (11, 19), as well as fewer targets for apatinib than anlotinib. TKIs have exhibited promising efficacy in the management of brain metastases. The necessity for agents to cross the blood-brain barrier to reach the CNS has limited the employment of several chemotherapeutics and targeted agents for CNS diseases. Whole-brain radiotherapy has been reported to be associated with a risk of neurotoxicity as well as stereotactic radiosurgery may be correlated with an increased risk of radionecrosis (20). Thus, there is an urgent need to develop novel and more effective drugs against brain metastases. Increasing evidence suggests the potential role of TKIs in controlling tumors within the CNS (21). Furthermore, results from the *post hoc* analysis of a phase III trial have suggested that anlotinib demonstrates activity in the brain and plays a potential role in the treatment of intracranial lesions (16). In this case, anlotinib has exhibited notable efficacy against brain metastases from breast cancer.

As of the data cut-off on March 2024, to the best of our knowledge, this is the first study to explore the efficacy and safety of anlotinib in the treatment of brain metastases from TNBC. In our study, the median CNS PFS was 7.2 months (95% CI, 3.5-10.9 months), and the median OS was 10.2 months (95% CI, 5.6-14.8 months). Moreover, the iORR and iDCR were 31.0% and 86.2%, respectively. These results indicated that anlotinib exhibited activity in the brain and had a positive impact on brain metastases from TNBC. Nevertheless, due to the limited availability of pertinent research, further investigations are required to gain a deepen knowledge of the specific mechanism of action. Furthermore, as a retrospective study, our study has limitations and still lacks the diagnosis of CNS injury at different moments of oncological treatment.

Few prior studies have addressed the treatment of brain metastases from breast cancer with anlotinib. In the study by Qian et al. on the efficacy and safety of anlotinib in metastatic breast cancer, 15 patients with brain metastases were included. The results showed that 7 patients achieved an SD, 5 patients had a PR, and 3 patients experienced a PD, with an ORR of 33%, a DCR of 80%, and a CNS PFS of 9.4 months, which were consistent with our findings (22). However, there was no distinction for TNBC in the study by Qian et al. Furthermore, the role of anlotinib in controlling various other intracranial tumors has been explored in several studies. The *post hoc* analysis of a phase III trial showed that anlotinib prolonged PFS and OS in non-small cell lung cancer (NSCLC) patients with brain metastases compared with placebo, while patients without brain metastases showed comparable improvements. The iORR was 14.3% and the iDCR was 85.7% in patients with brain metastases who were treated with anlotinib, indicating effective control of brain metastases. Additionally, anlotinib was associated with a higher incidence of neural toxicities (18.4% vs. 8.4%) and neurological symptoms (49.3% vs. 35.7%) than placebo, with no association with infarction or cerebral hemorrhage (16). Some studies and case reports have shown that the combination of anlotinib and radiotherapy may provide additional survival benefits in NSCLC patients with brain metastases. This combination therapy is effective in controlling both brain metastases and associated edema (23–27). Anlotinib can substantially reduce the proliferation of tumor microvessels, alleviate hypoxia in the tumor microenvironment, and enhance the efficacy of radiotherapy. The relief of hypoxia by anlotinib may result from vascular normalization, direct inhibition, or a combination of both (28–31). Early case reports have indicated that combination therapy with anlotinib and other drugs, including immune checkpoint inhibitors such as toripalimab and durvalumab, has proven to be effective in controlling brain metastases from small-cell lung cancer (32, 33). Moreover, anlotinib shows the ability of reducing or avoiding steroids and effectively mitigating cerebral edema caused by brain metastases (34). In the studies mentioned above, anlotinib has displayed an acceptable safety profile. Overall, these studies have shown that anlotinib is effective against brain metastases from lung cancer, suggesting its potential role in brain metastases from breast cancer. Some authors have found that the combination of anlotinib with other treatments can achieve better therapeutic effects on glioblastoma (35–37), suggesting the ability of anlotinib to penetrate the blood-brain barrier to act on intracranial tumors. Hence, we argued that in this retrospective cohort, anlotinib cross the blood-brain barrier and inhibit the growth of brain metastases, leading to a positive outcome from patients with brain metastases from breast cancer. Moreover, AEs that occurred during the treatment with anlotinib were acceptable in our study. However, a more detailed and precise understanding of the treatment mechanism requires further investigation.

## 5 Conclusion

The number of cases outlined in our description was relatively limited and the duration of anlotinib treatment is not entirely uniform, making the potential for some deviation in the data. However, based on the current study and other previous studies, it is evident that anlotinib holds significant promise in the treatment of brain metastases from TNBC. Moreover, anlotinib was generally safe and well tolerated, with a low incidence of treatment-related AEs.

## Data Availability

The original contributions presented in the study are included in the article/supplementary material. Further inquiries can be directed to the corresponding authors.
